# Supplementary material for: Effectiveness of Displaying Traffic Light Food Labels on the Front of Food Packages in Japanese University Students: A Randomized Controlled Trial
Source: Int J Environ Res Public Health. 2023 Jan 18;20(3):1806. doi: 10.3390/ijerph20031806 (PMC9914024; doi:10.3390/ijerph20031806)
Supplement: Supplementary file 1 [file ijerph-20-01806-s001.zip › ijerph-2031795-supplementary.pdf]

## Supplementary Material

**Table S1.** Bento Popularity Ranking Results and Nutrition Display Color Settings (Labeled Group).

| No | Bento popularity ranking | Tatal fat (g) | Satulated fat (g) | Sugar (g) | Salt (g) | Calorie (kcal) |
|---|---|---|---|---|---|---|
| 1 | Sukiyaki bento | 20.9 | 2.3 | 0.9 | 3.7 | 888 |
| 2 | Salt-grilled mackerel bento | 22.3 | 1.3 | 1.0 | 4.8 | 688 |
| 3 | Hamburger Steak Bento | 21.6 | 1.4 | 0.6 | 3.2 | 872 |
| 4 | Ginger grilled pork Bento | 22.2 | 3.0 | 0.4 | 3.9 | 899 |
| 5 | Grilled salmon bento | 21.1 | 0.5 | 0.9 | 2.8 | 644 |
| 6 | Grilled Meat Bento | 18.1 | 3.0 | 0.7 | 3.7 | 956 |
| 7 | Pork Cutlet Bento | 16.6 | 1.9 | 0.2 | 0.9 | 869 |
| 8 | Various Side Dishes Bento | 14.2 | 0.6 | 0.9 | 1.4 | 766 |
| 9 | Dried Laver Bento | 15.1 | 0.6 | 0.9 | 1.1 | 640 |
| 10 | Fried chicken bento | 16.4 | 0.9 | 1.0 | 0.7 | 734 |
| 11 | Fried oyster bento | 1.6 | 0.5 | 0.6 | 0.1 | 646 |
| 12 | Rice ball bento | 2.0 | 0.5 | 1.4 | 0.1 | 535 |
| 13 | Fried Prawn Bento | 2.0 | 0.5 | 0.6 | 0.1 | 676 |
| 14 | Fried Food Bento | 0.8 | 0.5 | 0.5 | 0.2 | 855 |
| 15 | Croquette bento | 2.1 | 0.7 | 0.3 | 0.2 | 761 |

The coloring of nutrition labels in dietary images was determined according to the university students' preferred diet, which was identified in advance via a dietary selection survey administered to them.In the Labeled group, TLF labels were displayed on the FOP of dietary images based on the above colors.
